# A Wafer Level Vacuum Encapsulated Capacitive Accelerometer Fabricated in an Unmodified Commercial MEMS Process

**DOI:** 10.3390/s150407349

**Published:** 2015-03-25

**Authors:** Adel Merdassi, Peng Yang, Vamsy P. Chodavarapu

**Affiliations:** 1 Department of Electrical and Computer Engineering, McGill University, McConnell Engineering Building, 3480 University Street, Montreal, QC H3A 0E9, Canada; E-Mail: adel.merdassi@mail.mcgill.ca; 2 CMC Microsystems, 945 Princess Street, Building 50, Innovation Park at Queen’s University, Kingston, ON K7L 3N6, Canada; E-Mail: yang@cmc.ca

**Keywords:** MEMS accelerometer, capacitive sensor, commercial MEMS process, low noise sensor, wafer-level vacuum encapsulation, Teledyne DALSA MIDIS process, inertial sensor

## Abstract

We present the design and fabrication of a single axis low noise accelerometer in an unmodified commercial MicroElectroMechanical Systems (MEMS) process. The new microfabrication process, MEMS Integrated Design for Inertial Sensors (MIDIS), introduced by Teledyne DALSA Inc. allows wafer level vacuum encapsulation at 10 milliTorr which provides a high Quality factor and reduces noise interference on the MEMS sensor devices. The MIDIS process is based on high aspect ratio bulk micromachining of single-crystal silicon layer that is vacuum encapsulated between two other silicon handle wafers. The process includes sealed Through Silicon Vias (TSVs) for compact design and flip-chip integration with signal processing circuits. The proposed accelerometer design is sensitive to single-axis in-plane acceleration and uses a differential capacitance measurement. Over ±1 g measurement range, the measured sensitivity was 1fF/g. The accelerometer system was designed to provide a detection resolution of 33 milli-g over the operational range of ±100 g.

## 1. Introduction

MEMS inertial sensors such as accelerometers and gyroscopes have become ubiquitous in our modern world [1,2]. Today, MEMS accelerometers have the second largest sales volume after pressure sensors and are used in a variety of applications including automotive, industrial, consumer electronics, and medical devices [3]. Each of above applications uses accelerometer sensors with different operational range from milli-g to several hundred g. In particular, high g (20 g and above) accelerometers are used in aerospace and automotive applications, oil & gas exploration, and structural health monitoring [4]. In the market today, many types of accelerometers are commercially available that are based on different operational principles including piezoresistance, piezoelectricity, optical, capacitive, and frequency resonance [5].

In the current work, we describe a single axis in-plane accelerometer sensor using capacitance transduction. Capacitive accelerometers are well suited for high g applications and offer low noise and low power operation. We are the first academic research group to implement an accelerometer sensor fabricated in an unmodified commercial MEMS process called, MEMS Integrated Design for Inertial Sensors (MIDIS), offered by Teledyne DALSA Semiconductor Inc. (TDSI, Bromont, QC, Canada) [6]. Development of MEMS sensors using a commercial MEMS foundry process allows highly efficient and reproducible manufacturing at very large volume, low cost, and extremely high yield [7,8]. The use of commercial MEMS foundry process ultimately enables the developed sensors to be commercially viable and successful. Many research groups have previously developed accelerometers in an unmodified commercial process including MUMPS [7,9], SUMMiT V [10], IMEC’s SiGe [11] and ThELMA [12].

Wafer level vacuum packaging of MEMS accelerometers has been demonstrated previously by several research groups [13,14]. We describe, for the first time, a MEMS accelerometer fabricated in a commercial MEMS process that itself includes wafer level vacuum encapsulation. Packaging technique and package-type currently plays one of the most important parameters for development of MEMS sensors such as accelerometers and can be a significant portion of the overall product cost [15]. Vacuum packaging is needed in the proper functioning and long term reliability of MEMS devices in avoiding stiction, controlling damping and limiting humidity exposure. Currently, low cost wafer-level or zero-level vacuum packaging techniques, that allow the use of high volume low cost plastic packaging at the device level, have become an important topic of research and development in the MEMS industry for a variety of devices including resonant sensors and inertial sensors [15,16].

In wafer-level or zero-level packaging, there are three important parameters for consideration that includes the sealing or encapsulation material, sealing leakage rate and chip-to-chip interconnection method [17,18,19]. The MIDIS process provides the current state-of-the-art solutions for the above three parameters that are suitable at mass-production scale. The MIDIS process allows high vacuum encapsulation at 10 milliTorr enabling very high Quality factor (*Q* factor) for the encapsulated devices. The total leak rate equivalent in the MIDIS process is 45 molecules/s (7.5E^−13^ atm∙cc/s) that is several orders of magnitude better than Military-Standard 883H (1E^−9^ atm∙cc/s or 61,500 molecules/s) that is commonly accepted by industry [6]. Finally, the MIDIS process includes sealed vertical Through Silicon Vias (TSVs) that minimize wastage of valuable die area and allow compact flip-chip integration with microelectronic signal processing circuits.

The MIDIS process is based on high aspect ratio bulk micromachining of 30 µm thick single-crystal silicon wafer that is vacuum encapsulated between two handle silicon wafers. The proposed accelerometer design is sensitive to in-plane single axis acceleration and uses a differential capacitive measurement with asymmetric configuration capable of detection resolution of 33 milli-g over the operational range of ±100 g. The proposed accelerometer uses strong U-hinges that help to improve performance by reducing the mechanical noise. The high aspect ratio of the fabricated springs enables high precision detection along single axis and avoids cross sensitivity from other axis that could generate a systematic error in sensor output. Like other capacitive inertial sensors, the proposed accelerometer is based on the principle of a proof mass undergoing an electrostatic force over the operating frequency range that is proportional to the input acceleration. The sensor readout circuit employed is a 24-bit 2-channel Σ-Δ capacitance-to-digital convertor (AD7746) available from Analog Devices Inc. (Norwood, MA, USA), that provides an I^2^C compatible output [20].

## 2. Accelerometer Sensor Structure

In this work, we present a comb-drive accelerometer that uses a proof mass, *m*, supported by two U-shaped springs or hinges with stiffness, *k*. The principle of working is based on static displacement proportional to the input acceleration over the operating frequency range. The displacement is then converted to an electrical output signal through differential capacitance transduction. Equation (1) gives the transmissibility function between the input acceleration, *a*, and the proof mass displacement, *x* [21]:

(1) $$\begin{array}{l} ma=m\overset{..}{z}+b\dot{z}+kz\\ \Rightarrow H(j\omega )=\frac{Z}{X}=\frac{\frac{\omega^{2}}{\omega_{n}^{2}}}{\sqrt{\left(1-\frac{\omega^{2}}{\omega_{n}^{2}}\right)+{\left(2\xi \frac{\omega }{\omega_{n}}\right)}^{2}}}\\ where\left\{\begin{array}{l} \xi =\frac{b}{2\sqrt{km}}\\ Z=Y-X \end{array}\right\} \end{array}$$

where, ξ, *b* and ω*_n_* are the damping ratio, damping coefficient, and natural angular frequency, respectively.

### 2.1. Mechanical Spring Structure

**Figure 1:**
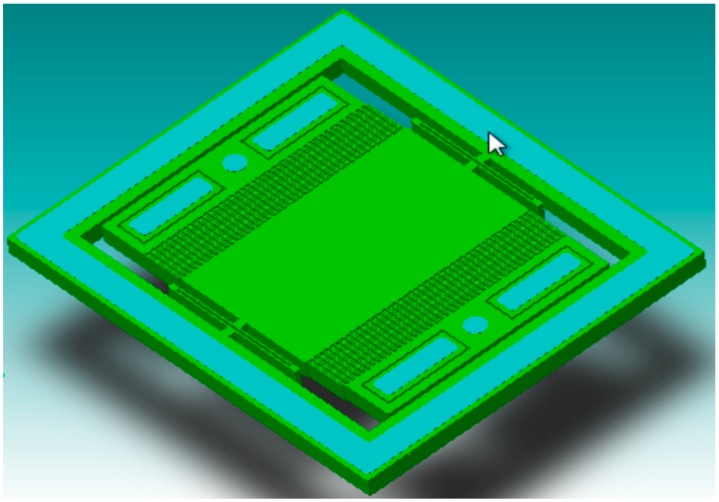
3-D Model of the single-axis capacitive accelerometer.

The proposed accelerometer is designed for high acceleration range of ±100 g. The proof mass has a rectangular shape to allow appropriate translational motion along its in-plane direction and its thickness is set by the fabrication process which is 30 µm. At low frequencies when *f << f_r_*, the mechanical sensitivity, *S_mec_*, is given by the Equation (2):

(2) $$S_{mec}=\frac{\Delta x}{\Delta a}=\frac{1}{\omega_{n}^{2}}$$

where, $\omega_{n}=\sqrt{\frac{k}{m}}$ is the natural angular frequency of the vibrating system. The springs supporting the proof mass are selected as U-shape that allows a better translation motion of rigid bodies. Figure 1 shows the 3D model of the device without the top sealing layer. The sensor includes springs with high stiffness that helps to reduce deflection due to the temperature variations. The parameters involved in the selection of the springs are given by Equation (3), where *E* is Young’s Modulus of Elasticity, *h* is spring beam thickness, *w* is spring beam width, *L* is spring beam length, *I_z_* is Moment of Inertia along *Z*-axis:

(3) $$K_{in-plane}=2K_{unit}=2\times \left(\frac{12EI_{z}}{L^{3}}\right)=2\times \left(\frac{Ew^{3}h}{L^{3}}\right)$$

In Equation (3), *h* and *E* are fixed by the MIDIS fabrication process. The values for *L* and *w* are optimized to obtain maximum deflection depending on the acceleration range, output linearity and the Design Rule Check (DRC) provided by TDSI for the MIDIS process. Further, all the corners are rounded in the two springs to avoid any fracture due to stress gradient and consequently to enhance the reliability of the device. The high aspect ratio of thickness to width for the springs helps to minimize disturbance from out of plane acceleration leading to low cross-axis sensitivity.

### 2.2. Electro-Mechanical Transduction

**Figure 2:**
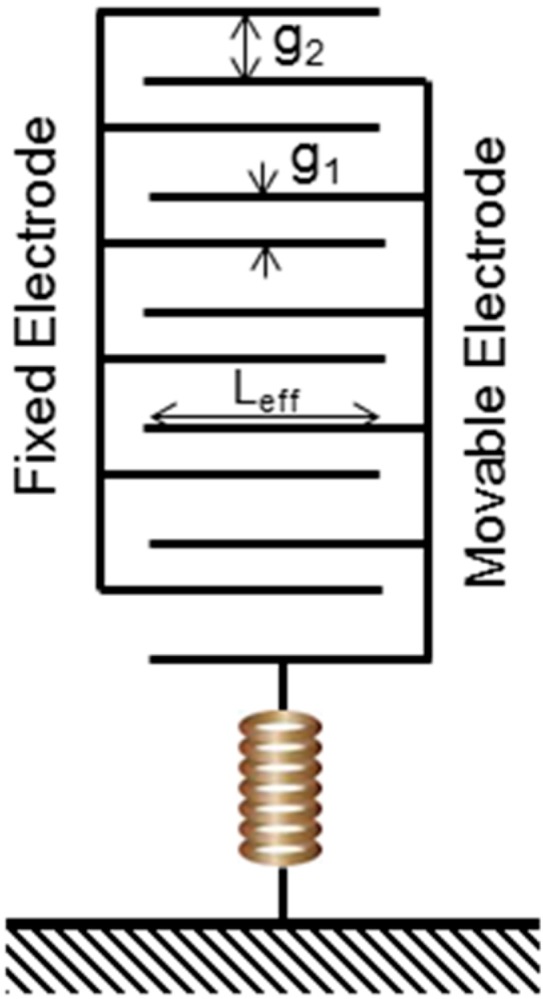
Inter-digitized comb fingers with asymmetric configuration.

The electrical transduction uses differential capacitance measurement through inter-digitized comb fingers with asymmetric configuration with two different gaps, *g*_1_ and *g*_2_. Figure 2 illustrates the asymmetric configuration of the transduction fingers with *g*_1_ > *g_2_*. For a small displacement, the electrical sensitivity, *S_ele_* can be approximated by the Equation (4). In our sensor, the initial capacitance value of the sensor structure is around 1.7 pF with Δ*C*_max_ of 320 fF. The assumed capacitance values are affected by parasitic capacitances after physical device implementation. In our sensor, the optimal ratio between *g*_1_ and *g*_2_ is determined to be 6:

(4) $$S_{ele}=\frac{\Delta C}{\Delta x}=C_{0}\frac{g_{2}-g_{1}}{g_{2}g_{1}}$$

The overall sensitivity of the sensor is deduced from the multiplication of Equations (3) and (4) for *S_ele_* and *S_mec_*, respectively, and is given by Equation (5):

(5) $$S_{sensor}=\frac{\Delta C}{\Delta a}=\frac{C_{0}\Delta g}{\omega_{n}^{2}g_{2}g_{1}}$$

### 2.3. Improved Noise Performance

There are three techniques to improve the accelerometer signal to noise ratio, *a_n_*, as given by Equation (6), where, *k_B_*, *T*, *K_spring_* are the Boltzmann constant, operating temperature, and spring stiffness, respectively. First, the *Q* factor can be increased by reducing the damping factor. In the present case, this is done through the wafer level packaging under high vacuum of 10 milliTorr. Second, we employed the maximum allowable gap of 30 µm, between the moving structure and the capping layer in order to decrease the damping coefficient. Finally, the mechanical noise was minimized by selecting rigid springs with higher spring stiffness, *K_spring_*, without drastically reducing the mechanical sensitivity [22]:

(6) $$a_{n}=\sqrt{\frac{4k_{B}T\omega_{n}}{mQ}}$$

Typically, the main sources of damping effect that decrease the *Q* factor is due to two mechanisms, the first is related to the squeeze air film confined in the inter-digitized fingers [22] and the second concerns the slide air film between the sealing cap and the moving plate. Both these factors are negligible in the proposed sensor structure due to wafer level packaging under high vacuum of 10 milliTorr.

The magnitude of electrical noise is reduced by using Σ-Δ based capacitance to digital converter (CDC) [20]. The AD7746 CDC circuit from Analog Devices offers low power consumption and low electrical noise. It uses a combination of two signal processing techniques, namely, the oversampling 24-bit Σ-Δ modulator and noise shaping filtering. Therefore, the Total Noise Equivalent Acceleration, *TNEA*, is given by Equation (7) [22]:

(7) $$TNEA=\sqrt{a_{n}^{2}+{\left(\frac{\delta C}{S_{sensor}}\right)}^{2}}$$

where, δ*C* is the effective minimum capacitance detectable by the conditioning circuit per √Hz.

## 3. Simulation Results and Discussion

**Figure 3:**
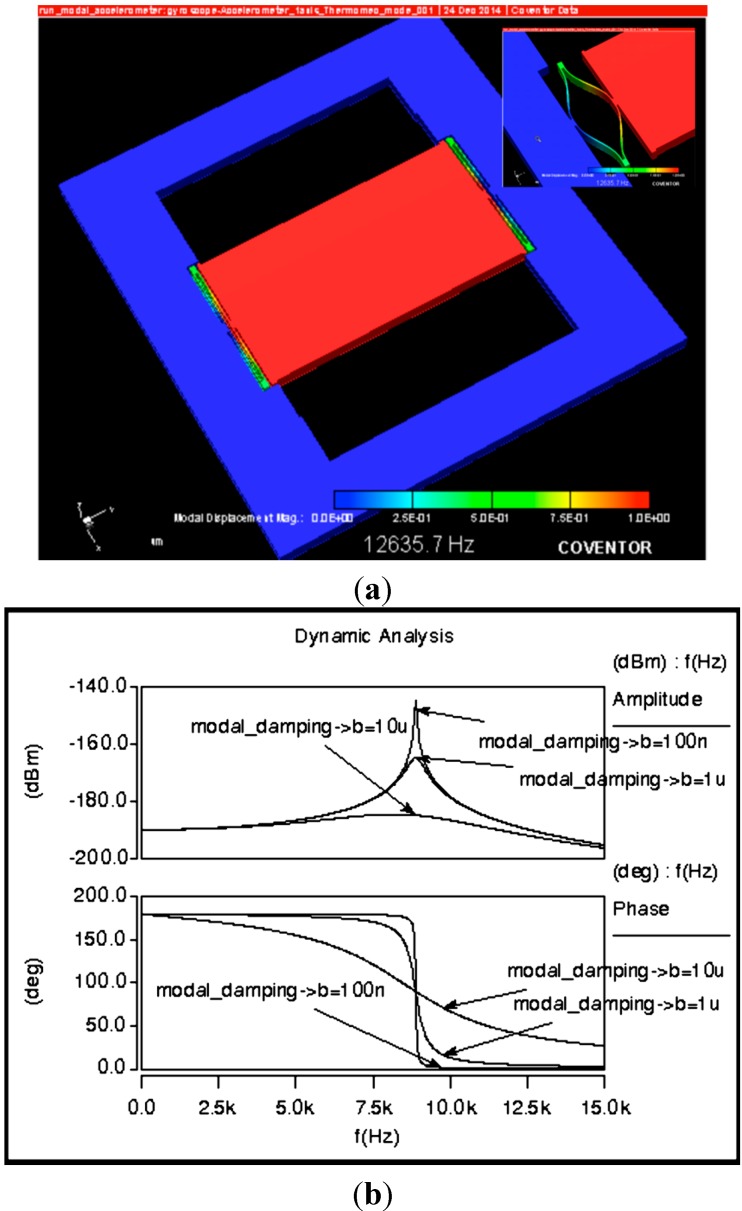
Device modeling results. (**a**) FEM modeling; (**b**) Lumped modeling.

The simulation results are based on Finite Element Modeling (FEM) using Coventorware software for electro-mechanical, modal and damping coefficient simulations. We performed lumped modeling using Architect module where full integration of sensor and its signal conditioning circuit is implemented for time domain analysis. The modal analysis is used to show the dynamic characteristics of the accelerometer. The first mode, which illustrates the resonant frequency along the sensitive *Y*-axis gives the full frequency range over which the sensitivity can be considered as constant. Figure 3a illustrates the FEM modeling for the first mode at 12.6 kHz with an exaggerated illustration in the upper right corner. However, using lumped modeling, the frequency analysis shows the amplitude and the phase of the sensitive axis with a resonant frequency around 9.5 kHz as illustrated by Figure 3b. The difference in the results is mainly due to the comb fingers that add more mass to the proof plate. Also, Architect uses a model of perfect beam that gives a more accurate result than FEM method. In our sensor structure, the maximum displacement is fixed at 15% from the initial gap g_1_ in order to insure a good linearity.

**Figure 4:**
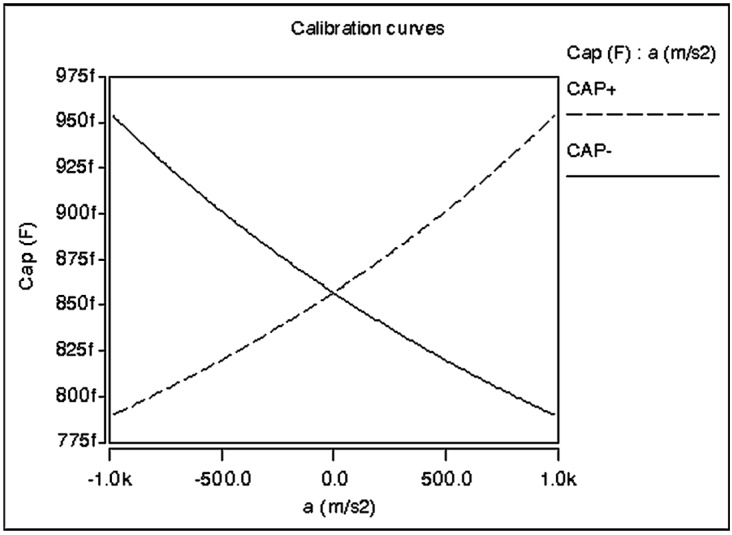
Electro-mechanical analysis showing the calibration curve response.

The capacitance measurement is based on differential measurement in order to double the total capacitance change and consequently improving the sensor sensitivity. The initial capacitance value for the accelerometer is selected to be 1.7 pF. Figure 4 shows the calibration curve of the accelerometer sensor for both polarities of the differential capacitance.

**Figure 5:**
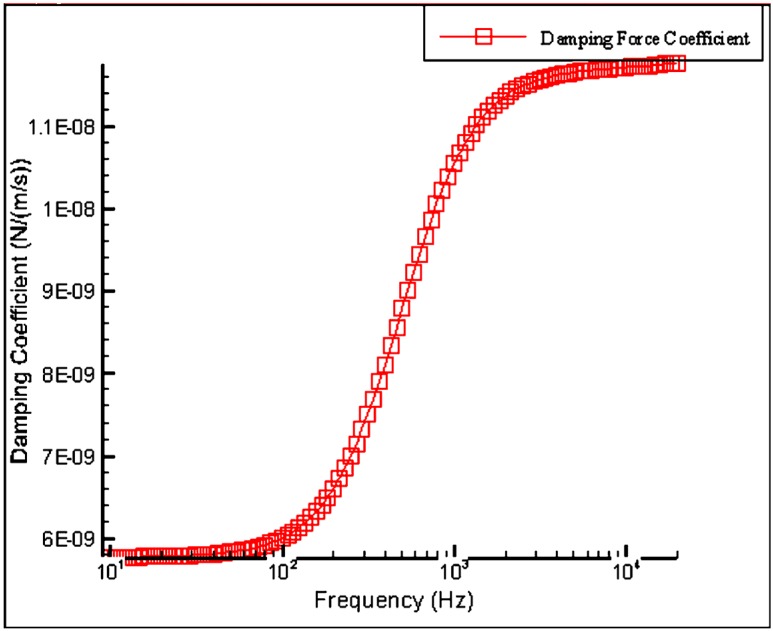
Simulation of damping force coefficient over the operational frequency range.

We aim to deduce the damping force coefficient in condition of high vacuum. Here, two simulations were performed for the slide film effect and stokes fluid effect. Figure 5 illustrates the variation of the damping force coefficient in terms of operating frequency. At resonance frequency, the value of the damping force coefficient is around 1.17 × 10^−8^ (N/m/s). The damping coefficient in stokes flow reaches 3.42 × 10^−7^ (µN/µm/s), which means that the effect of inter-digitized fingers on damping is more significant than the slide flow mechanism.

## 4. Experimental Results and Discussion

**Figure 6:**
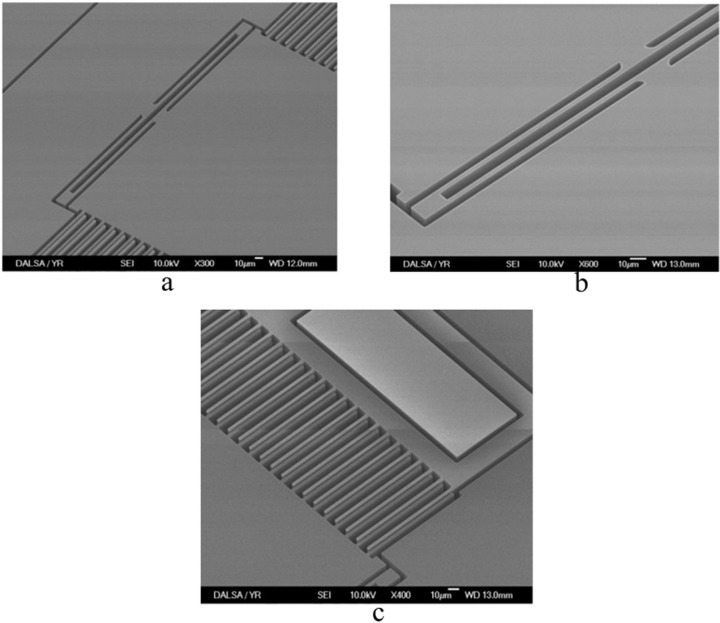
SEM images of the fabricated accelerometer device. (**a**) Accelerometer sensor; (**b**) U-shaped springs; (**c**) Inter-digitized sensing electrodes.

The accelerometer device is fabricated using MIDIS process available from TDSI [5]. The Scanning Electron Microscope (SEM) image of the fabricated accelerometer is shown in Figure 6a that shows the proof mass suspended by U-shaped mechanical springs. Figure 6b shows the SEM image of spring structure with its rounded corners and Figure 6c shows the close-up view of the comb-drive electrodes. Table 1 shows the chosen specifications for the accelerometer device. The experimental measurements aim to deduce the sensor sensitivity and the associated resolution which is affected by the measured total noise. Here, the sensor data is recorded by the 24-bit 2-channel Σ-Δ capacitance-to-digital convertor (AD7746) available from Analog Devices Inc. that is maintained at room temperature (23 °C). The Σ-Δ modulator helps to significantly minimize the noise and therefore helps to enhance the measurement accuracy.

**Table 1:** Accelerometer specifications.

Parameters	In-Plane Metrics
Mechanical Sensitivity	2.83 × 10^−8^ (m/m/s^2^)
Sensor Sensitivity	1.6 fF/g
Resolution	33 mg
Resonance frequency	9.45 (kHz)
Mechanical Acceleration Noise	2.23 × 10^−2^ (µg∙√Hz)
Dimensions	350 × 600 × 30 (µm^3^)

**Figure 7:**
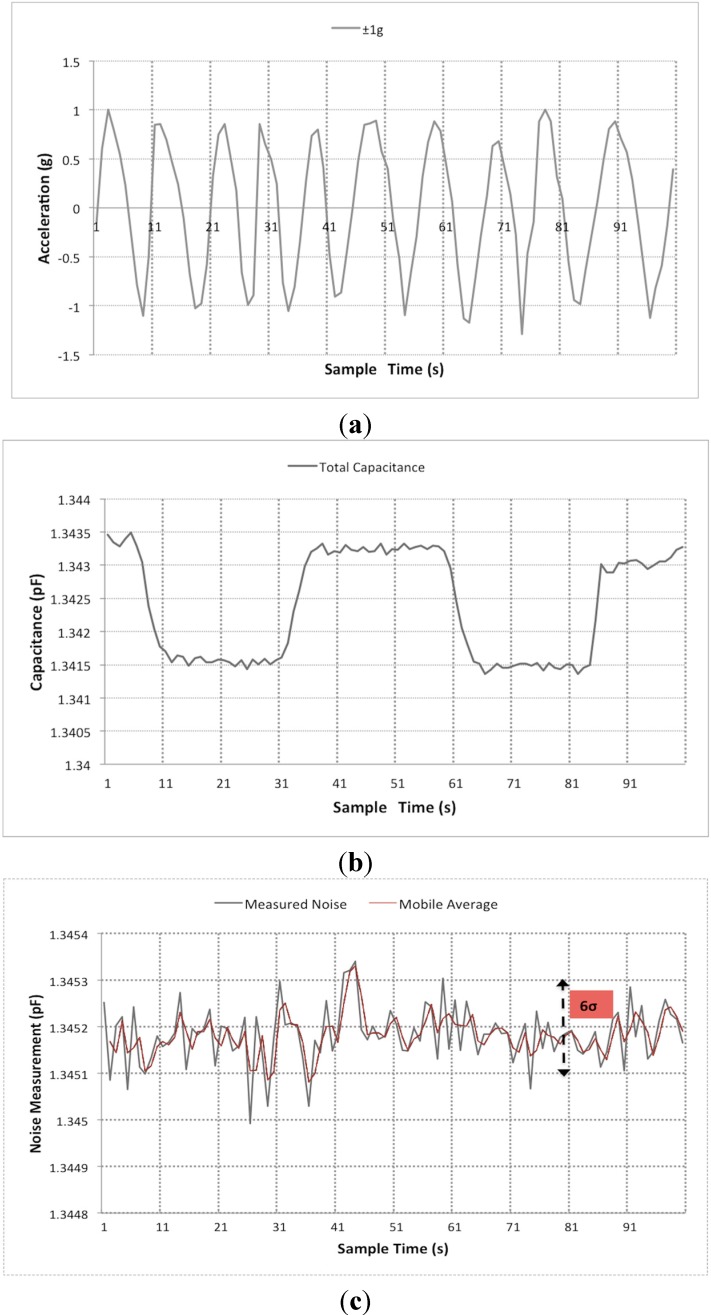
Experimental measurement results. (**a**) Response from ±1 g acceleration; (**b**) Capacitance measurement over ±1 g; (**c**) Generated noise from sensor system.

The measurements consist of manually tilting the chip by 180° repeatedly over 10 times to change the acceleration due to gravity from +1 g to −1 g as shown in Figure 7a. Over the ±1 g measurement range, the sensitivity was measured as 1 fF/g from Figure 7b, which is close to 1.6 fF/g as noted from simulation results and the experiment was repeated over two cycles. The maximum noise measured corresponds to the minimum detectable signal and consequently determines the resolution of the sensor. This parameter is extracted by dividing the full range by 6σ as shown in Figure 7c, which leads to a resolution of 33 milli-g.

## 5. Conclusions

We report, for the first time, the design and fabrication of a MEMS accelerometer sensor in an unmodified commercial MEMS process that includes wafer-level vacuum encapsulated. Specifically, the accelerometer sensor was fabricated in MIDIS process recently introduced by Teledyne DALSA Semiconductor Inc. The proposed accelerometer design is sensitive to in-plane single axis acceleration and uses a differential capacitive measurement. Our approach allows highly efficient and reproducible manufacturing at very large volume, low cost, and extremely high yield. Over ±1 g measurement range, the measured sensitivity was 1 fF/g. The accelerometer system was designed to provide a detection resolution of 33 milli-g over the operational range of ±100 g.
